# Hybrid versus total sublaminar wires in patients with spinal muscular atrophy undergoing scoliosis surgery

**DOI:** 10.1186/s12891-021-04737-0

**Published:** 2021-10-11

**Authors:** Shih-Hsiang Chou, Wen-Wei Li, Cheng-Chang Lu, Kun-Ling Lin, Sung-Yen Lin, Po-Chih Shen, Yin-Chun Tien, Hsuan-Ti Huang

**Affiliations:** 1 Department of Orthopaedic Surgery, Kaohsiung Medical University Hospital, Kaohsiung Medical University, Kaohsiung, Taiwan; 2 Orthopaedic Research Center, Kaohsiung Medical University, Kaohsiung, Taiwan; 3 Regeneration Medicine and Cell Therapy Research Center, Kaohsiung Medical University, Kaohsiung, Taiwan; 4 Departments of Orthopedics, School of Medicine, College of Medicine, Kaohsiung Medical University, Kaohsiung, Taiwan; 5 Department of Orthopaedics, Kaohsiung Municipal Siaogang Hospital, Kaohsiung Medical University, Kaohsiung, Taiwan; 6 Department of Obstetrics and Gynecology, Kaohsiung Medical University Hospital, Kaohsiung Medical University, Kaohsiung, Taiwan; 7 Department of Obstetrics and Gynecology, School of Medicine, College of Medicine, Kaohsiung Medical University, Kaohsiung, Taiwan; 8 Department of Orthopedics, Kaohsiung Medical University Hospital, Kaohsiung Medical University, Kaohsiung, No. 100, Shiquan 1st Rd., Sanmin Dist., Kaohsiung City, Taiwan

**Keywords:** Spinal muscular atrophy, Segmental instrumentation, Sublaminar wire, Hybrid constructs, Scoliosis

## Abstract

**Background:**

Early versions of spinal muscular atrophy (SMA) scoliosis correction surgery often involved sublaminar devices. Recently, the utilization of pedicle screws has gained much popularity. Pedicle screws are generally believed to provide additional deformity correction, but pedicle size and rotational deformity limit their application in the thoracic spine, resulting in a hybrid construct involving pedicle screws and sublaminar wire. Studies of the efficacy of hybrid instrumentation in SMA scoliosis are often limited by the scarcity of the disease itself. In this study, we aimed to compare the surgical outcomes between hybrid constructs involving pedicle screws and sublaminar wire and sublaminar wire alone in patients with SMA scoliosis.

**Methods:**

We retrospectively reviewed the clinical records and radiographic assessments of patients with SMA scoliosis who underwent corrective surgery between 1993 and 2017. The radiographic assessments included deformity correction and progressive changes in the major curve angle, pelvic tilt (PT) and coronal balance (CB). The correction of deformities was observed postoperatively and at the patient’s 2-year follow-up to test the efficacy of each type of construct.

**Results:**

Thirty-three patients were included in this study. There were 14 and 19 patients in the wiring and hybrid construct groups, respectively. The hybrid construct group demonstrated a higher major curve angle correction (50.5° ± 11.2° vs. 36.4° ± 8.4°, *p* < 0.001), a higher apical vertebral rotation correction (10.6° ± 3.9° vs. 4.8° ± 2.6°, *p* < 0.001), and a reduced progression of the major curve angle at the 2-year follow-up (5.1° ± 2.9° vs. 8.7° ± 4.8°, *p* < 0.001). A moderate correlation was observed between the magnitude of correction of the apical vertebral rotation angle and the major curve (r = 0.528, *p* = 0.002).

**Conclusion:**

This study demonstrated that hybrid instrumentation can provide a greater magnitude of correction in major curve and apical rotation as well as less major curve progression than sublaminar wire instrumentation alone in patients with SMA scoliosis. Level of evidence III

**Supplementary Information:**

The online version contains supplementary material available at 10.1186/s12891-021-04737-0.

## Introduction

Spinal muscular atrophy (SMA) is an autosomal recessive neuromuscular disease characterized by a progressive course of muscular weakness and atrophy. Scoliosis is the most common orthopaedic manifestation in these patients, and its incidence is positively correlated with the severity of SMA. Individuals with type 1 or type 2 SMA almost always present with scoliosis, while only approximately 50% of individuals are affected in type 3 SMA [1].

The deformities involved are often located in both the thoracic and lumbar spine, with a collapsing “C”-shaped curve with associated obliquity of the pelvis [2, 3]. Loss of coronal and sagittal balance makes it difficult to maintain an upright posture, and the rapid deterioration of pulmonary function greatly affects the quality of life for both the patient and the caregiver [3]. Conservative management with bracing is rarely used as a definitive treatment because of its minimal efficacy and potential constrictive effects on the already compromised respiratory system [4]. Surgical treatment is usually indicated in the early stage of life, even before skeletal maturity, to control the progression of the deformity and to preserve cardiopulmonary function [5, 6].

Surgical correction of neuromuscular scoliosis was first performed through Harrington’s distraction rod, and there has been considerable advancement since then [7]. Modern scoliosis surgeries are mostly based on the concept of segmental instrumentation and fusion, as described by Luque, and are used in combination with various pelvic fixations [2, 8, 9]. Early versions of posterior instrumentation often involved the use of sublaminar devices, e.g.*,* wires, bands, and hooks. Recent iterations often involve pedicle screws in place of or in combination with wiring for segmental fixation [9, 10]. The kickstand rod technique emerged as an intelligent way to achieve correction in both the coronal and sagittal planes in adult spinal deformities [11].

There are few comparative studies on the surgical outcomes of neuromuscular scoliosis surgery [9, 10, 12–14]. Reportedly, the full pedicle screw method provides better correction of the major curve than the hybrid method in scoliosis surgery for cerebral palsy patients [10]. Comparable surgical results between sublaminar wire, hybrid and pedicle screw methods in scoliotic surgery for cerebral palsy and Duchenne muscular dystrophy patients have also been reported [9, 12, 14]. However, studies comparing deformity correction in SMA using different methods remain scarce. In this study, we aimed to retrospectively compare the clinical and radiological outcomes of sublaminar wires and those of hybrid constructs consisting of pedicle screws and sublaminar wires in patients with SMA undergoing surgery for scoliosis.

## Methods

### Participants

After Institutional Review Board approval was obtained, the medical records and radiographic assessments of patients with SMA scoliosis who underwent surgical correction using either total sublaminar wire constructs or hybrid constructs including sublaminar wire in the high thoracic spine and pedicle screws in the low thoracic and lumbar spine between 1993 and 2017 were retrospectively analysed by two paediatric orthopaedic surgeons. For all patients, the diagnosis of SMA and the associated neuromuscular scoliosis was confirmed based on clinical manifestations and radiographic examinations by paediatric neurologists. All surgeries were performed by the same paediatric orthopaedic surgeon.

### Surgical procedure

The surgical indications for SMA scoliosis include pulmonary function deterioration, progressive scoliosis (major curve angle > 40°), and difficulty sitting. All the reviewed patients underwent surgery using a segmental spinal construct either with the total sublaminar wire construct or with the hybrid construct (sublaminar wire and pedicle screw) as described in our previous report [6]. The construct extended from T2 or T3 to the sacrum, and the Galveston pelvic fixation technique was used in all cases [6]. In the hybrid construct group, the insertion of pedicle screws was attempted until the pedicle size reached the screw limit. Cross-link systems were used for augmentation in every case. Pedicle screws were inserted into the apical vertebrae if feasible.

### Postoperative course

Every patient was transferred to the paediatric intensive care unit for intensive care by a paediatric cardiopulmonologist immediately after the surgery. Patients were observed for 24 h after extubation and then transferred to the general ward for further postoperative care and rehabilitation. Boston braces were used for at least three months to support successful bone fusion. Once bone fusion occurred, especially in the lumbar-pelvic area, bracing was no longer recommended.

### Assessments

Radiographic parameters, including major curve angle, pelvic tilt (PT), and coronal balance (CB), were measured using Cobb’s method and our previously described method on anteroposterior and lateral sitting radiographs of the entire spine [15]. Radiographic parameters from preoperative, postoperative, and 2-year follow-up radiographs were measured and recorded.

To obtain a unified assessment of apical vertebral rotation across all enrolled patients, the rotation angle of the apex vertebra on the transverse plane was calculated with the method developed by Chi et al. using anterior-posterior X-ray images [16]. The surgical time was defined as the time from skin incision to completion of wound closure. Complications, including stroke, deep vein thrombosis, pulmonary complications, and renal failure, were also recorded.

### Statistical analysis

Descriptive analyses were performed for each parameter. The Wilcoxon test was used to compare the demographic profiles, surgical parameters, and radiological profiles. Pearson correlation analysis was performed between the amount of correction for each pair of radiological profiles, and their corresponding correlation coefficient (r) was calculated. All analyses were performed with the Statistical Package for the Social Sciences (version 19.0, SPSS, Inc., Chicago, IL).

## Results

**Fig. 1:**
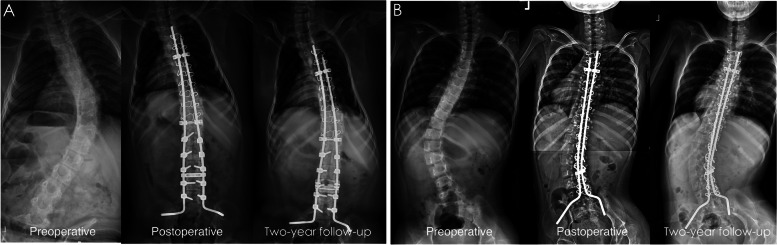
Representative preoperative, postoperative and two-year follow-up anterior-posterior radiographs of patients who received the (**A**) hybrid construct and (**B**) total sublaminar wire construct

Thirty-three patients were enrolled in this study. There were 14 and 19 patients in the sublaminar wire group and the hybrid instrumentation group, respectively. There was no significant difference between the two groups in terms of demographic data (Table 1) or preoperative radiographic parameters (Table 2). There were 10 (71%) SMA II and 4 (29%) SMA III patients in the sublaminar wire group and 12 (63%) SMA II and 7 (37%) SMA III patients in the hybrid instrumentation group. In the hybrid instrument group, sublaminar wires were mostly used in the high thoracic vertebrae due to the limitations of the pedicle size, and lumbar or thoracolumbar pedicle screws were applied if feasible (Fig. 1).

**Table 1:** Demographic profiles of patients with SMA undergoing instrumentation and fusion

	Sublaminar wire (*n* = 14)	Hybrid (*n* = 19)	*P* value
Demographic profile			
Male: Female	9:5	10:9	0.723
SMA II: SMA III	10:4	12:7	0.719
Age (years)	14.5 ± 5.5	14.5 ± 9.4	0.981
Height (cm)	144.2 ± 11.1	151.7 ± 12.4	0.101
Weight (kg)	32.5 ± 9.4	42.0 ± 14.5	0.058
Operation duration (hours)	8.3 ± 1.4	9.1 ± 1.5	0.109

All data are presented as the mean ± standard deviation

**Table 2:** Radiographic parameters of patients with SMA undergoing instrumentation and fusion

	Sublaminar wire (*n* = 14)	Hybrid (*n* = 19)	*P* value
Major curve angle			
Preoperative (°)	58.8 ± 18.6	69.8 ± 19.0	0.107
Postoperative (°)	22.4 ± 13.4	19.3 ± 12.5	0.491
Correction (°)	36.4 ± 8.4	50.5 ± 11.2	< 0.001*
Correction (%)	63.8 ± 11.7	74.1 ± 10.5	0.013*
2-year follow-up	31.1 ± 17.3	25.9 ± 14.2	0.347
Major curve progression (°)	8.7 ± 4.8	5.1 ± 2.9	0.011*
Pelvic tilt (°)			
Preoperative (°)	10.7 ± 6.8	15.9 ± 9.6	0.092
Postoperative (°)	5.6 ± 3.5	6.7 ± 4.4	0.432
Correction (°)	5.1 ± 4.3	9.2 ± 7.3	0.073
Correction (%)	45.2 ± 19.2	55.8 ± 20.7	0.146
2-year follow-up	7.0 ± 4.3	8.2 ± 5.1	0.469
Pelvic tilt progression (°)	1.4 ± 1.2	1.5 ± 1.2	0.833
Coronal balance (cm)			
Preoperative (°)	5.4 ± 2.8	5.2 ± 2.2	0.897
Postoperative (°)	2.6 ± 1.8	2.2 ± 0.9	0.474
Correction (°)	2.8 ± 1.9	3.0 ± 1.7	0.674
Correction (%)	50.8 ± 21.6	53.0 ± 17.8	0.756
2-year follow-up	3.1 ± 2.0	2.9 ± 1.1	0.657
Coronal balance progression (°)	0.5 ± 0.3	0.7 ± 0.4	0.355

All data are presented as the mean ± standard deviation

The radiographic results are shown in Table 2. The correction of the major curve was significantly better in the hybrid instrumentation group than in the sublaminar wire group (50.5° ± 11.2° vs. 36.4° ± 8.4°, *p* < 0.001). The correction magnitude of the PT was higher for the hybrid construct group but not to a statistically significant degree (9.2° ± 7.3° vs. 5.1° ± 4.3°, *p* = 0.073). There was no significant difference in CB correction between the two groups (*p* = 0.674). The hybrid constructs not only provided a better correction to the major curve immediately after surgery but also had a lower rate of scoliosis progression at the 2-year follow-up (5.1° ± 2.9° vs. 8.7° ± 4.8°, *p* = 0.011). The progression in the PT and the CB were similar in both groups at the 2-year follow-up (*p* = 0.833 and *p* = 0.355, respectively).

The most common level of the apical vertebra in both groups was the L1 vertebra, followed by the L2 vertebra (Table 3). No significant difference was noted between the amounts of apical vertebral rotation before the index surgery of the two groups. The hybrid instrumentation provided a significantly greater magnitude of transverse rotation correction than the sublaminar wire (10.6° ± 3.9° vs. 4.8° ± 2.6°, *p* < 0.001) (Table 3). Comparing the surgical outcome of patients who received pedicle screw placement in the apical vertebra (9 cases) and those without apical screw placement (10 cases) in the hybrid instrumentation group, there was no significant difference in the magnitude of correction (e.g., major curve, PT, and CB) between subgroups (Supplementary Table S1).

**Table 3:** Apical vertebral distribution and apical vertebral rotation angle of patients with SMA undergoing instrumentation and fusion

	Sublaminar wire (*n* = 14)	Hybrid (*n* = 19)	*P* value
Apical vertebral location: number of patients	L1:8 L2:6	T9:1 T11:1 L1:14 L2:3	
Apical vertebral rotation angle			
Preoperative (°)	18.1 ± 6.6	18.8 ± 5.7	0.756
Postoperative (°)	13.3 ± 5.6	8.2 ± 4.1	0.005
Correction (°)	4.8 ± 2.6	10.6 ± 3.9	< 0.001
Correction (%)	28.1 ± 13.1	57.6 ± 17.5	< 0.001

All data are presented as the mean ± standard deviation

Correlations between each of the abovementioned radiological parameters were performed using Pearson correlation analysis. A moderate correlation was observed between the magnitude of correction of the apical vertebral rotation angle and the major curve (r = 0.528, *p* = 0.002) (Table 4). A very weak level of correlation was found between the other radiological parameters. Specifically, the correlation coefficient suggests little to no correlation between the PT and CB.

**Table 4:** Pearson correlation analysis between the magnitudes of correction of radiological parameters

	AVRA	Major curve	Pelvic tilt	Coronary balance
AVRA	1			
Major curve	0.528	1		
Pelvic tilt	0.119	0.330	1	
Coronary balance	0.113	0.193	−0.005	1

*AVRA* apical vertebral rotation angle

During the follow-up, one patient developed pseudoarthrosis, and cephalad wire migration was found in three patients. Iliac bone osteolysis was initially noted in most patients, which might have been due to the enlarging procedure for rod insertion or a windshield wiper motion of the rod. Solid bone formation was achieved in all patients in their later follow-ups.

## Discussion

In this study, we demonstrated that the hybrid constructs can provide better correction power for the major curve angle and apical vertebral rotation as well as a lower rate of major curve progression at the 2-year follow-up than sublaminar wiring. This could be largely related to the 3 column fixation and increased derotation ability of the pedicle screws in hybrid instrumentation relative to the sublaminar wires [17], which provide mostly coronal plane correction and a very limited ability to fixate length and derotate. To the best of our knowledge, this is the largest cohort study solely focused on patients with SMA that demonstrated the advantage of hybrid instrumentation over sublaminar wiring in SMA scoliosis correction. Although there are limited studies on SMA scoliosis correction, comparative studies between the sublaminar wire and hybrid instrumentation for other neuromuscular scoliosis corrections also indicated similar results (Table 5) [9, 10, 12–14]. In studies of cerebral palsy patients [9, 10, 12], pedicle screw constructs achieved shorter surgical times and intraoperative blood loss amounts but better major curve correction than hybrid constructs. Using sublaminar bands in the hybrid construct helped achieve similar correction rate outcomes to those of all-pedicle screw constructs. Arun et al. reported that there were similar clinical and radiographic outcomes in Duchenne muscular dystrophy scoliosis surgery between sublaminar wire, hybrid and pedicle screw constructs, but a longer operating time and more blood loss were reported in the sublaminar wire group [14]. Moreover, the authors emphasized the importance of early detection of curves and the need for early surgery to prevent extended instrumentation use and intraoperative complications.

**Table 5:** Comparative studies on the surgical correction of neuromuscular scoliosis

	Current study	Wimmer et al. [12]	Watanabe et al. [13]	Arun et al. [14]	Mattila et al. [10]	Albert et al. [9]
No. cases	33	52	44	43	66	29
Major population (n)	SMA (33)	CP (17)	N (N)	DMD (43)	CP (31)	CP (17)
Study design	Sublaminar wire vs. hybrid instrumentation	Sublaminar wire vs. hybrid instrumentation	Wire vs. hook vs. anterior screw vs. pedicle screw	Sublaminar wire vs. hybrid vs. pedicle screw	Hybrid instrumentation vs. pedicle screw	Hybrid instrumentation^a^ vs. pedicle screw
Average follow up (months)	24	42.9	48	56.4	33.6	29
Surgical outcome on neuromuscular scoliosis.	Better curve correction, less blood loss and less loss of major curve correction in hybrid instrumentation group.	Comparable surgical results and satisfactory questionnaires between the two fixation methods (Luque-Galveston/Isola-Asher system).	Pedicle screws had the greatest correction rate, the smallest loss of correction and the greatest amount of correction of the apical vertebral translation in scoliotic curves greater than 100°.	Comparable results during medium- to long-term follow up for all methods. Longer operating time and more blood loss in the sublaminar wire group.	Pedicle screw group had the shortest operating times, the least blood loss and the best correction of the major curve	Sublaminar bands utilized in a hybrid construct can achieve corrections equivalent to all-pedicle screw constructs

Abbreviations: *SMA* spinal muscular atrophy, *CP* cerebral palsy, *N* not specified, *DMD* Duchenne muscular dystrophy

^a^sublaminar band and pedicle screw

The value of hybrid instrumentation with sublaminar wiring in the thoracic spine is unique for paediatric scoliosis correction, especially in the Asian population. Total pedicle screw instrumentation and fusion is currently a mainstay treatment in scoliosis surgery. However, Asians have been demonstrated to have a smaller pedicle diameter (T4 vertebral, 2.9 ± 1 mm) than the Caucasian population (T5 vertebral, 4.7 mm) [18, 19], with the mean pedicle size from T3 to T9 below 4 mm. In addition, poor nutritional status in combination with the fact that most neuromuscular scoliosis patients receive surgical intervention during early adolescence further limits the size of the pedicle and increases the difficulty of pedicle screw insertion. Inserting pedicle screws in the upper thoracic spine is not only technically demanding but also increases the risk of neurovascular complications [14]. Moreover, the low bone mineral density of patients with SMA is another potential risk for breaching the cortical wall during pedicle screw placement [20], which may result in early internal fixation failure and nerve injury [21, 22]. On the other hand, hybrid instrumentation has been shown to provide correction power comparable to total pedicle screw instrumentation for other flaccid types of neuromuscular scoliosis [9, 14]. Similar results have also been reported between other hybrid instrumentations and total pedicle screw instrumentation in patients with adolescent idiopathic scoliosis [23–25]. These results suggest that hybrid instruments could be a possible alternative for ethnic groups with a smaller build if total pedicle instrumentation and fusion are not feasible.

Throughout our study, all our patients underwent the Galveston procedure for pelvic fixation, and the magnitude of PT correction and the correction maintained were similar across different groups. The Galveston procedure provided superior resistance to flexion forces and can achieve a fusion rate between 88 and 95% [26, 27]. Its low instrument profile provides the additional benefit of a lower chance of wound dehiscence [26]. Recently, its application has decreased because of its technical difficulty and the popularity of other spinopelvic fixation techniques, e.g., iliac screws and S2-alar-iliac screws. Despite its decreased popularity, the Galveston procedure has been shown to provide greater pullout strength [28] and a similar wound complication rate with respect to iliac screws [29], thus remaining the method of choice in our institution. Coronal balance is strongly related to postoperative pain, disability and functional outcome [30]. In our study, both groups of patients underwent segmental fixation with two-rod methods and obtained good correction in the coronal plane. The implantation of supplementary rods, called kickstands, is an innovative method that provides powerful tridimensional forces, allowing for coronal and sagittal imbalance correction in spine deformity patients [11].

All patients in this series received one-stage posterior instrumented correction surgery instead of an extra anterior procedure. This uniform posterior approach avoided the comorbidities of the anterior procedure, and the creation of a single skin scar can help produce to a greater aesthetic outcome [31]. From our previous study, we observed that patients who underwent correction surgery obtained good sitting balance and tolerance [6]. Pulmonary function was maintained after surgery during long-term postoperative follow-up. Therefore, early detection of severe scoliotic curves in SMA patients or even in patients with rare syndromic diseases such as Kleefstra syndrome [32] is important because surgery for spine deformities could help improve their posture and improve the quality of life.

Our results showed a moderate correlation between the correction of the major curve angle and apical vertebra derotation in patients who underwent SMA scoliosis correction. Derotation manoeuvres were found to result in greater major curve correction in adolescent idiopathic scoliosis, but the correlation between coronal correction and apical vertebral derotation has not been reported [33, 34]. Comparable surgical outcomes were noted between patients who received apical pedicle screws and those who did not in our study. This suggests that apical derotation can be achieved with periapical instrumentation and that apical screws are not necessary for the construct, as previous studies demonstrated that concave apical screws were not related to additional correction power [24, 35].

The limitations of this study included its limited sample size due to the scarcity of the disease itself. Additionally, some of our medical records dated back to more than two decades ago, when computed tomography was not included as the routine imaging modality for scoliosis surgery. This limits our ability to determine the rotational deformity of each vertebral body. The surgical techniques have been modified and have evolved over time, which increased the heterogeneity of the correction results. Nevertheless, this study provided a good demonstration of major curve correction and derotation with hybrid constructs in scoliosis surgery for patients with SMA.

## Conclusion

In this study, we demonstrated that hybrid instrumentation can provide a greater magnitude of major curve correction and better correction maintenance than sublaminar wire instrumentation in patients with SMA scoliosis. Given the limitation of pedicle diameter and vertebral rotation, hybrid constructs can serve as an alternative surgical intervention for patients with SMA undergoing scoliosis corrective surgery.

## Supplementary Information

**Additional file 1.**

## Data Availability

The datasets generated and/or analysed during the current study are not publicly available due to *privacy*/ethical restrictions but are available from the corresponding author on reasonable request.
