# Supplementary material for: Hybrid versus total sublaminar wires in patients with spinal muscular atrophy undergoing scoliosis surgery
Source: BMC Musculoskelet Disord. 2021 Oct 11;22:867. doi: 10.1186/s12891-021-04737-0 (PMC8507395; doi:10.1186/s12891-021-04737-0)
Supplement: Supplementary file 1 — Additional file 1. [file 12891_2021_4737_MOESM1_ESM.docx]

**Supplementary Table S1.** Comparison of radiographic parameter of patients with

Hybrid group with or without instrumented screw at apex level.

|  | **Patient with apex instrumented screw (n=9)** | **Patient without apex instrumented screw (n=10)** | **P value** |
| --- | --- | --- | --- |
| **Major curve angle** |  |  |  |
| Preoperative (°) | 66.9 ± 15.5 | 72.4 ± 22.1 | 0.542 |
| Postoperative (°) | 16.9 ± 6.4 | 21.4 ± 16.2 | 0.447 |
| Correction (°) | 50.0 ± 10.6 | 51.0 ± 12.2 | 0.852 |
| Correction (%) | 75.3 ± 5.9 | 73.0 ± 12.7 | 0.627 |
| **Pelvic tilt (**°**)** |  |  |  |
| Pre-operative (°) | 15.4 ± 12.1 | 16.4 ± 7.4 | 0.826 |
| Postoperative (°) | 5.6 ± 3.6 | 7.7 ± 4.9 | 0.310 |
| Correction (°) | 9.7 ± 9.6 | 8.6 ± 4.9 | 0.748 |
| Correction (%) | 60.0 ± 22.7 | 52.0 ± 19.1 | 0.415 |
| **Coronal balance (cm)** |  |  |  |
| Preoperative (°) | 5.5 ± 2.1 | 5.0 ± 2.4 | 0.681 |
| Postoperative (°) | 2.0 ± 0.8 | 2.3 ± 1.0 | 0.499 |
| Correction (°) | 3.4 ± 1.6 | 2.7 ± 1.8 | 0.363 |
| Correction (%) | 59.4 ± 13.0 | 47.2 ± 20.2 | 0.141 |

All data are presented as the mean ± standard deviation.
